# Advances in Mineral Nutrition Transport and Signal Transduction in Rosaceae Fruit Quality and Postharvest Storage

**DOI:** 10.3389/fpls.2021.620018

**Published:** 2021-02-22

**Authors:** Qian Bai, Yuanyue Shen, Yun Huang

**Affiliations:** Beijing Key Laboratory for Agricultural Application and New Technique, College of Plant Science and Technology, Beijing University of Agriculture, Beijing, China

**Keywords:** mineral nutrition, Rosaceae fruit, fruit development, quality formation, postharvest storage

## Abstract

Mineral nutrition, taken up from the soil or foliar sprayed, plays fundamental roles in plant growth and development. Among of at least 14 mineral elements, the macronutrients nitrogen (N), potassium (K), phosphorus (P), and calcium (Ca) and the micronutrient iron (Fe) are essential to Rosaceae fruit yield and quality. Deficiencies in minerals strongly affect metabolism with subsequent impacts on the growth and development of fruit trees. This ultimately affects the yield, nutritional value, and quality of fruit. Especially, the main reason of the postharvest storage loss caused by physiological disorders is the improper proportion of mineral nutrient elements. In recent years, many important mineral transport proteins and their regulatory components are increasingly revealed, which make drastic progress in understanding the molecular mechanisms for mineral nutrition (N, P, K, Ca, and Fe) in various aspects including plant growth, fruit development, quality, nutrition, and postharvest storage. Importantly, many studies have found that mineral nutrition, such as N, P, and Fe, not only affects fruit quality directly but also influences the absorption and the content of other nutrient elements. In this review, we provide insights of the mineral nutrients into their function, transport, signal transduction associated with Rosaceae fruit quality, and postharvest storage at physiological and molecular levels. These studies will contribute to provide theoretical basis to improve fertilizer efficient utilization and fruit industry sustainable development.

## Introduction

Many species of Rosaceae are rich in economic value, such as apple, pear, peach, apricot, plum, cherry, loquat, mango, almond, and strawberry. Fruits are rich in vitamins, sugars, and organic acids, as well as many kinds of health-benefit compounds (Mahmood et al., 2012). These compounds, including anthocyanins and vitamins, may have anti-inflammatory and antioxidant effects, and thus fruits are usually used to maintain a balanced diet (Kumari et al., 2017). In addition, dry fruits, such as almond, are especially abundant in nutrition and also are widely cultivated in the world (Maguire et al., 2004). Fruits not only can be eaten directly but also can be used for food industry in the production of wine, juice, jam, and so on. Thus, fruits are important components in our life, and improvement of fruit quality is going on all the time in orchard management.

It is well known that mineral nutrients, especially macronutrients nitrogen (N), phosphorus (P), potassium (K), and calcium (Ca) as well as micronutrient iron (Fe), are essential for plant growth, fruit yield, and quality (Falchi et al., 2020; Singh et al., 2020). In recent years, many studies have strengthen our insights into the influence and the molecular mechanisms for mineral nutrition (N, P, K, Ca, and Fe) in Rosaceae plant growth, fruit development, quality, and preservation, especially highlighting N in fruit yield and P and K in fruit quality. Mineral nutrition, such as N, P, and Fe, not only affects fruit quality directly but also affects the absorption of other nutrient elements.

In this review, we integrated many previously defined signaling components linked to mineral nutrition transport and signal transduction in the regulation of fruit quality at physiological and molecular levels. This review will not only contribute to provide the theoretical basis to improve fertilizer efficient utilization and fruit industry sustainable development but also highlight the important aims of future work.

## Two Faces of Nitrogen Application in Fruit Yield and Quality

Nitrogen (N) is a ubiquitous element that constituted nucleic acids, amino acids, proteins, chlorophyll, as well as many other metabolites in plants. Therefore, it is an indispensable important nutrient element for plant growth and development. Plants acquire inorganic N from the soil, mainly in the form of ammonium and nitrate, and low or excessive N level has a serious influence on fruit yield and quality.

When strawberries were treated with three levels of NO_3_^−^ (9, 12, and 15 mol/m^3^), the highest NO_3_^−^ concentration of nutrient solution produced the maximum yield, but the nutraceutical quality, such as antioxidant capacity and phenolic compounds, significantly declined (Preciado-Rangel et al., 2020). Under sufficient soil moisture, the application of N fertilizer to potted apple trees caused an increase in stomatal conductance, which decreased water use efficiency (WUE; Qu et al., 2000). Under soil drought, the WUE of apple trees under nitrogen fertilizer was significantly higher than that of the control, owing to the promotion of mesophyll capacity and photosynthesis (Qu et al., 2000).

As the increasing of N supply in apple (*Malus domestica* Borkh.) trees, the concentrations of sucrose (Suc), glucose (Glc), fructose (Fru), and total nonstructural carbohydrates (TNC) decreased (Cheng et al., 2004), resulting in the production of organic acids. It is confirmed that in strawberries, applying high nitrogen amounts could lead to an increase in citric acid content and conceal fruit sweet flavor, whereas low nitrogen levels could decrease acidity and result in an observably lower content of ascorbic acid and higher fruit firmness (Cardeñosa et al., 2015).

Anthocyanin and chlorophyll are remarked signs for fruit quality, and many research studies suggest that increasing N supply decreases anthocyanin synthesis but induces chlorophyll content in fruits. Under high N nutrition in apple trees, shoot growth was enhanced, and the activity of phenylalanine ammonia lyase, a key enzyme involved in flavonoid biosynthesis, seemed to be downregulated, resulting in a generally decreased flavonoid accumulation (Strissel et al., 2005). N fertilization could be used to suppress anthocyanin formation in green apple cultivars, such as “Granny Smith,” in that red blush was undesirable (Ritenour and Khemira, 2007). High N could lead to higher chlorophyll concentration of fruit skin, thus increasing fruit greenness; on the contrary, nitrate deficiency suppressed nitrate absorption and assimilation and inhibited the transformation of 5-aminolevulinic acid (ALA) into porphobilinogen (PBG), thus suppressing chlorophyll synthesis in apple (Wen et al., 2019). Peach fruit from the higher N rates was greener and had higher total soluble solids than fruit from the trees under low N (Rubio Ames et al., 2020).

The application of the nitrification inhibitor, 3,4-dimethylpyrazole phosphate (DMPP) and abscisic acid (ABA), could effectively improve the problem of poor fruit coloring caused by excessive N fertilizer application used in apple trees (Wang et al., 2020a,b). DMPP reduced the capacity of N absorption and N accumulation in fruits and whole apple plant, whereas it increased fruit anthocyanin and soluble solid content (SSC), and it had no significant effect on fruit yield (Wang et al., 2020a). An appropriate ABA concentration could promote the expression of anthocyanin synthesis genes and transcription factors, as well as the anthocyanin content of the “Red Fuji” apple peel. More than that, exogenous ABA reduced the accumulation of N and increased the contents of sugar and carbon in fruits (Wang et al., 2020b).

To improve the anthocyanin content and accordingly the quality and market value of important agricultural commodities, it can be a powerful tool to supply nutrient strategically. In particular, the precise application of N fertilizer should not only improve fruit yield but also positively influence fruit quality including anthocyanin content (Jezek et al., 2018).

### Molecular Mechanisms of Nitrogen Assimilate, Transport, and Action

Thirteen ammonium transporters of *AMT1* subfamily in the apple rootstock *Malus robusta* Rehd were grouped into four clusters: clade I (*MrAMT1;1* and *MrAMT1;3*), clade II (*MrAMT1;4*), clade III (*MrAMT1;2*), and clade IV (*MrAMT1;5*). All the *AMT1s*, except *MrAMT1;4*, were highly expressed in vegetative organs and significantly responded to different nitrogen supplies. The ammonium absorption activities of these *AMT1s* were confirmed in the AMT-defective mutant yeast system, though their NH_4_^+^ uptake kinetics varied (Li et al., 2017).

There were 73 NPF (Nitrate Transporter 1/Peptide Transporter Family) genes in apple that were organized into eight major groups (Wang et al., 2018b). *MdNPF6.5* was strongly induced by both low-nitrate and high-nitrate levels, and the overexpression of *MdNPF6.5* in apple calli enhanced the tolerance to low-N stress, suggesting that MdNPF6.5 has greater nitrogen uptake activity (Wang et al., 2018b). Under low N treatment, the expression of nitrate transporter *MdNRT2.4* is higher in high nitrogen-use efficiency (NUE) apple cultivars than in low NUE apple (Wang et al., 2019a), which may explain the NUE differences among apple cultivars.

Anion channel PbrSLAH3 of pear (*Pyrus bretschneideri*) could rescue the ammonium toxicity phenomenon of *slah3* mutant grown in high-ammonium/low-nitrate conditions, which was activated by phosphorylation by calcium-dependent protein kinase PbrCPK32 (Chen et al., 2019b). The overexpression of autophagy proteins *MdATG18a* and *MdATG9* enhanced the tolerance to N-deficiency stress and the Suc concentration in apple callus (Sun et al., 2018b; Huo et al., 2020). MdATG18a played a role in nitrate uptake and assimilation by the upregulation of nitrate reductase (NR) MdNIA2 and nitrate transporters MdNRT2.1/2.4/2.5 (Sun et al., 2018b). MdATG9 regulated the expression of *MdNRT1.1*, *MdNRT2.5*, *MdNIA1*, and *MdNIA2* in response to N starvation in apple (Huo et al., 2020). Overexpressing *MdHY5* (transcription factor LONG HYPOCOTYL 5) in apple callus induced the expression of N acquisition-related genes and increased the activity of NR, finally improving nitrate contents (An et al., 2017).

## Phosphorus Fertilizer Improves Fruit Yield, Quality, and Postharvest Storage Quality

Phosphorus (P) supplementation has become a primary agricultural management aspect of fruit production, because P not only affects fruit yield but also modulates the production of the soluble solid and secondary metabolites, such as ascorbic acid and flavonoids, which are closely related to fruit quality.

When appropriate phosphorus was added, the weight of strawberry fruits increased notably (Afroz et al., 2016). The SSC was lower in fruits from P shortage strawberries, indicating that P deficiency could negatively affect sugar biosynthesis (Valentinuzzi et al., 2015a). SSC in strawberry fruits was positively related to the phosphorus content, and P fertilizer could effectively increase SSC in strawberry fruit (Cao et al., 2015). Meanwhile, P content, WUE, and photosynthetic rate (Pn) increased in phosphoric acid-treated strawberry fruits (Cao et al., 2015).

P starvation resulted in a significant change of bioactive compounds in strawberry fruits. The contents of vitamin C, malic acid, galactaric acid, proline, lysine, sorbitol-6-phosphate, malate, and citrate were also inhibited when P was deficient (Valentinuzzi et al., 2015b; Afroz et al., 2016; Zhang et al., 2020), whereas anthocyanin contents were higher (Valentinuzzi et al., 2015a). However, field experiments had shown that the application of phosphite (Phi), a salt of phosphorous acid absorbed and transferred in a way similar to inorganic P, increased the anthocyanin content of strawberry (Estrada-Ortiz et al., 2013). Therefore, the effect of fruit quality formation varies with the type and timing of P fertilizer application (Jezek et al., 2018).

P deficiency also changes other ion contents in plants. The fruit firmness of strawberry fruits under P deficiency increased 60% compared with that of the control, which may be because of the higher concentration of Ca (Valentinuzzi et al., 2015a). Moreover, low P alleviated the Fe deficiency phenotype in apples and improved the ferric-chelated reductase activity of the rhizosphere, because P shortage promotes proton exocytosis and organic acids exudation and enhances Fe absorption, thus increasing the Fe content in both the shoots and roots under Fe deficiency in apples (Zhang et al., 2020). For another, the limited elongation of the main roots under low inorganic phosphate (Pi) conditions may be results of the Fe accumulation in the apical meristem (Zhang et al., 2020).

Many plants can utilize phosphate from places outside the nutrient-depleted zone through symbiotic arbuscular mycorrhizal fungi (AMF; Ezawa and Saito, 2018). The colonization by AMF and appropriate P supplementation of traditional (TF) or organic (OF) fertilizer was regarded as the main approach to raise the yield and quality of strawberry fruits in acidic soils with low P availability (Parada et al., 2019). There was a highly positive correlation between the concentration of P in apple rootstocks and the bacterial genera *Bacillus* (Chai et al., 2020). The P-efficient hybrid apple lines had better growth characteristics and stronger root structure, as well as more abundant *Bacillus* in the rhizosphere, than the P-inefficient hybrid line plants (Chai et al., 2020). However, according to some reports, there is no correlation between the AMF association and low P tolerance when plants are grown under optimal nutrient conditions (Cockerton et al., 2020).

Trisodium phosphate (TSP) treatment could maintain the postharvest quality of apple fruit by inhibiting respiration intensity, delaying weight loss, and inhibiting the decline of flesh firmness, ascorbic acid, titratable acid, and SSC (Ge et al., 2019). The TSP treatment also delayed the decrease of the content of ADP, ATP, and energy charge in apple fruit (Ge et al., 2019).

### Molecular Pathways of Phosphate Signal in Rosaceae Species

Roots uptake Pi from the soil mainly by PHT1 family transporters that are located in the plasma membrane (Shin et al., 2004; Wang et al., 2020c). The PHT2-type carriers are plastid envelope-located, and the PHT3-type carriers are inner mitochondrial membrane-located (Rausch and Bucher, 2002). Trans-Golgi-located PHT4;6 proteins function as transporters that can release Pi from the Golgi apparatus (Guo et al., 2008; Hassler et al., 2012). The free phosphate is mainly stored in the plant vacuole, and the Arabidopsis VPT1 (PHT5;1) proteins have been identified as vacuolar Pi influx transporters (Liu et al., 2015). OsVPE1 and OsVPE2 are responsible for *Oryza sativa* vacuolar Pi efflux (Xu et al., 2019).

Phosphate starvation response 1 (PHR1) is a key transcription factor involved in Pi-starvation signaling binding to a cis-element named PHR1-binding sequence (P1BS) and significantly regulates Pi starvation-induced (PSI) genes, including Pi transporters (*PHT1*) and *miR399* (Chiou and Lin, 2011). *miR399* could suppress the expression of ubiquitin-conjugating E2 enzyme PHO2 and therefore release PHT1 and PHO1, involved in phosphate transduction from root to shoot, thus promoting Pi acquisition and transport in Arabidopsis (Chiou and Lin, 2011). However, only a few phosphate transporters and signal transduction components were reported in Rosaceae species.

A total of 37 putative phosphate transporters in apple were identified into five clusters (MdPHT1, MdPHT2, MdPHT3, MdPHT1, and MdPHT5; *M. domestica*), and their expression was tissue specific and significantly regulated by low P treatment (Sun et al., 2017). The overexpression of *MdMYB2* could regulate the expression levels of PSI genes and further promote the assimilation and utilization of phosphate in apple (Yang et al., 2020). The levels of SUMOylation and *MdSIZ1*, a (SUMO) E3 ligase encoding gene, were induced by Pi-deficient conditions, suggesting that *MdPHR1* and *MdMYB2* might be targets for the SUMO protein (Zhang et al., 2019a).

Some important components involved in phosphate transport and signal regulation have been reported in strawberry. The expression level of *FaPHO1;H9*, homologous with *PHO1* from *Arabidopsis thaliana*, was related to P content in fruits during different developmental stages and under different P fertilizer supplements (Cao et al., 2017). The overexpression of *FvPHR1* and *FvmiR399a* in woodland strawberries significantly increased fruit quality (Wang and Wu, 2017; Wang et al., 2019b). FvPHR1 was found to directly bind to the promoter of *FvmiR399a* and positively regulate its expression (Wang et al., 2019b). PHR1-miR399 participates in the phosphate signaling pathway and maintains the phosphorus homeostatic state of woodland strawberry (Wang and Wu, 2017; Wang et al., 2019b). A tonoplast phosphate transporter FaVPT1 induced by Suc was identified to regulate fruit ripening and quality in strawberry (Huang et al., 2019). The overexpression of *FaVPT1* in strawberry fruits promoted ripening and increased the contents of P, sugar, and anthocyanin, decreased fruit firmness, and affected the expression of ripening-related genes (Huang et al., 2019).

## Appropriate Potassium Fertilizer Improves Fruit Yield, Quality, and Plant Stress Resistance

Potassium (K) affects the growth of plant and fruit yield, quality, and nutrient content of apple, strawberry, pear, sweet cherry, and almond (Kumar and Ahmed, 2014; Lu et al., 2015; Wang et al., 2018b; Zhang et al., 2018b; Shen et al., 2019; Hüseyin and Ömer, 2020; Preciado-Rangel et al., 2020). Furthermore, the application of K fertilizer at different periods may affect utilization efficiency. For example, 50% K fertilizer at post-anthesis + 50% at the expansion stage decreased the content of Ca in “Fuji” apple fruits significantly, which was detrimental to fruits postharvest storage (Lu et al., 2015). In the orchard, the best period for K fertilizer application was the basal and expansion period equally (Lu et al., 2015).

Fruits from K fertilizer application treatment had higher SSC, and the activities of sugar metabolic enzymes significantly increased in apple (Zhang et al., 2018b). The biomass and K^+^ content of root decreased significantly of pear seedlings was affected by K deficiency treatment (Wang et al., 2018c). Appropriate K supply promoted the transport of nutrients and sugar, by which increased the content of sugar in “Huangguan” pear fruits (Shen et al., 2019).

K fertilizer application treatment not only promotes greater fruit quality but also affects the utilization of nitrogen and the transport of other cations. The deficit or excess of K inhibited the uptake and utilization of nitrogen (N), whereas appropriate K supply could promote photosynthesis, enhance the activity of NR, and then increase the nitrogen absorption of *Malus hupehensis* (Tian et al., 2017; Xu et al., 2020). Ca^2+^ concentration increased with increasing K fertilization levels during apple fruit development (Zhang et al., 2018b). The Mg transporters increased under low K, whereas these decreased under medium and high K in pear tree, indicating that Mg had an obvious compensation effect on K, and K had an obvious antagonistic effect on Mg (Shen et al., 2019).

Elevated external K fertilization supply alleviated both biological and abiotic stresses in fruit trees (Araujo et al., 2015; Song et al., 2015a; Peng et al., 2016). K fertilization supply enhanced antioxidant defense systems, by which reducing Zn toxicity of peach seedlings (Song et al., 2015a). Increasing K content in apple trees enhanced resistance to *Valsa* canker, one of the most destructive diseases of apple, pathogen colonization, and establishment, so improved management of K fertilization could effectively control the disease incidence and development of *Valsa* canker (Peng et al., 2016). Spraying the plants with K Phi would induce the phenylpropanoid pathway of mango plants to reduce internal necrosis and the disease development of *Ceratocystis fimbriata* (Araujo et al., 2015).

### Molecular Pathways of K Transporters and Channels in Rosaceae Species

K^+^ usability, uptake efficiency, and transfer efficiency remarkably affect the performance of plants. In order to maintain optimal K^+^ homeostasis, more than 71 K^+^ transporters and channels, including three channel families and three transporter families, have already been identified in *A. thaliana* (Wang and Wu, 2017; Wang et al., 2020c). They are involved in K^+^ absorption by roots, ion transport between organs and tissues, and K^+^ storage in vacuoles (Gierth and Mäser, 2007; Chanroj et al., 2012; Gomez-Porras et al., 2012).

Selective K^+^ channels contain three families: Shaker, TPK, and Kir-like. The Shaker K^+^ channels display six transmembrane segments (TMS). TPK channels (Tandem-Pore K^+^ Channels) display a hydrophobic core composed of four TMS. Plant Kir-like channels are associated with animal Kir (K^+^ inward rectifier) channels, displaying two TMS. There are three different families of plasma membrane K^+^ transport systems: the HAK/KUP/KT K^+^ transporters (Gierth and Mäser, 2007), the HKT high-affinity K^+^ transporters (Rodríguez-Navarro and Rubio, 2006), and the CPA2 subfamily, including K^+^ efflux (KEA) H^+^/K^+^ antiporters and the cation/H^+^ exchanger (Mäser et al., 2001).

Some K^+^ channels and transporters have been reported, but there is far less information in Rosaceae species. Thirty-six Shaker K^+^ channel genes from Rosaceae species were divided into five subgroups, including a K^+^ channel protein PbrKAT1 that may play a role in regulating salt tolerance of pear, because its activity was inhibited by external sodium ions (Chen et al., 2019a). Similarly, a strawberry K^+^ channel, FaKAT1, serves as a positive regulator in the regulation of fruit ripening in an ABA-dependent manner (Song et al., 2017). K^+^ channel AKT1 is activated and phosphorylated by calcineurin B-like protein (CBL)-interacting protein kinases (CIPKs; Xu et al., 2006). *FaAKT1* encoded a highly K^+^-selective channel and showed upregulated expression in strawberry “Camarosa” upon salt stress (Garriga et al., 2017). The overexpression of *FaTPK1*, a strawberry tonoplast K channel, promoted the expression of fruit ripening-related genes and the contents of anthocyanin, soluble sugars, and ABA (Wang et al., 2018c). FaTPK1 could not only regulate fruit ripening and quality formation but also enhance fruit resistance to *Botrytis cinerea* (Wang et al., 2018c).

Sixteen KT/HAK/KUP family K transporters in peach (*Prunus persica*) were identified and highly expressed in fruit and flower, indicating that these transporters may play important roles in K^+^ uptake and transport as well as fruit development in peach (Song et al., 2015b). *PbrHAK1* and *PbrHAK12/16* were obviously increased under K^+^ deficiency, suggesting their significant roles in K^+^ uptake of pear, especially in response to K^+^ starvation (Wang et al., 2018d). HKT genes and CPA genes were identified in five Rosaceae species, involving *Fragaria vesca*, *Pyrus communis*, *M. domestica*, *P. persica*, and *Prunus mume*, and *HKT* genes of woodland strawberry were responding to the salt stress (Zhang et al., 2019b). FaHKT1 could transport Na^+^ selectively, and the increased expression of *FaHKT1* in roots correlates with the higher tolerance to salinity of the strawberry genotype (Garriga et al., 2017). CPA genes of pear were mainly expressed in pollen tubes and fruits, which suggested that CPAs may play significant roles in the growth of pollen tubes (Zhou et al., 2016).

Signal network of K regulating fruit enlargement and ripening was studied in “Huangguan” pear (Shen et al., 2017). K contents in the pear leaves and fruits were significantly reduced under low-K treatment. According to the transcriptome sequencing data, AKT1 and HAK/KUP/KT genes may play a vital role on K^+^ transport in leaves and fruits under K deficiency. High K level could promote photosynthesis and modify the distribution of the carbohydrate and nutrient from leaves to fruits, whereas carbohydrate metabolism was inhibited by low K during maturation. Two Suc synthase (SUS) genes were remarkably downregulated in fruits, and this change may be the reason for the decrease Suc concentration of leaves and fruit under low K. Genes associated with ethylene, cytokinin (CK), jasmonic acid (JA), auxin, and ABA were induced by low K, but the genes encoding transporters of auxin, CK, and brassinosteroid were significantly downregulated under low-K treatment in leaves and fruit (Shen et al., 2017).

Studying the signal network of different K applications on plant growth and fruit development and the molecular mechanism of K application on improving fruit quality can provide scientific basis for improving fruit quality by reasonable fertilizer.

### Calcium Fertilizer Regulates Fruit Ripening and Postharvest Quality

Calcium (Ca) is considered to be one of the most important mineral elements to determine fruit quality, and many research studies focus on the function of Ca. Studies have confirmed that Ca has a central role in cell wall interactions, plant signaling, and water relations (Hocking et al., 2016). Since Ca is an important part of cell wall structure, it can affect the integrity of the cell membrane as well as affect a key role in membrane function (Fallahi et al., 1997).

Localized Ca deficiencies observed in particular species or varieties can lead to leakage of membranes, irregular softening of the cell walls, and abnormal development of fruit. It is reported that high concentrations of Ca inhibited ethylene production in apple slices, by delaying the conversion of 1-aminocyclopropane-l-carboxylic acid (ACC) to ethylene (Lieberman and Wang, 1982; Han et al., 2020). The de-esterification of pectin and Ca crosslinking resulted in the changes of the physical properties of the cell wall, which eventually lead to fruit softening (Hocking et al., 2016).

Ca deficiencies in fruits may also cause pathological and physiological disorders (Hocking et al., 2016). Ca^2+^ deficiency contributed to the occurrence of skin browning spot, which is an important physiology disorder in Huangguan (*P. bretschneideri* × *Pyrus pyrifolia*) pear fruit (Dong et al., 2015). The Ca^2+^ imbalance of pear fruit would lead to the development of hard end disorder (Wang et al., 2018e). Calcium application during sweet cherry development and ripening caused a reduction in respiration rate and water-induced fruit cracking, which was accompanied by numerous changes in the polar/non-polar primary and secondary metabolites according to the metabolome profiling (Michailidis et al., 2020).

Abnormal Ca content, partitioning, and distribution are the major factors related to bitter-pit (BP; De Freitas et al., 2015). Almost all factors that influence BP incidence in preharvest fruits could be directly or indirectly associated with Ca content in apple fruit (Ferguson et al., 1999). It is studied that the main reason for the decrease of BP of apple (*M. domestica*) is the change of K/Ca ratio (Guerra and Casquero, 2010). Ca-containing fertilizers, such as CaCl_2_, Ca(NO_3_)_2_, or Ca(HCOO)_2_, were applied in preharvest apple, in order to improve fruit quality, because as the Ca content increased, the BP was reduced (Yu et al., 2018).

Ca-containing substances are widely used to maintain the quality of fruit after harvest. Treatment with CaCl_2_ or ultrasound and calcium (U + Ca) boosted fruit total phenolics content in strawberry by stimulating the genes expression involving anthocyanin structure (Xu et al., 2014), preventing the decrease in firmness of strawberries to maintain better fruit quality (Zhang et al., 2018a). Sprays of CaCl_2_ and Ca(NO_3_)_2_ effectively reduced respiration rate, spoilage, and physiological weight loss, but maintained fruit firmness, palatability, acidity, and pectin methylesterase (PME) activity during storage of peach, apple, apricot, sweet berry, and plum fruits, which helped fruits to be stored longer with acceptable edible quality (Gupta, 2011; Mauro et al., 2016; Liu et al., 2017; Michailidis et al., 2019; Sinha et al., 2019; Han et al., 2020). The combination of calcium propionate (CP) and sodium chlorite (SC) could increase the firmness of apple slices and inhibited apple browning and the growth of *Escherichia coli* and yeast on fresh-cut apple slices (Guan and Fan, 2010). However, it is recently reported that postharvest calcium dipping, including CaCl_2_ and Ca(NO_3_)_2_ treatment, in apples caused an increase in lenticel breakdown, the damage during storage appearing as discrete tissue deterioration surrounding the lenticels (Singh et al., 2016, 2021).

Ca nutrition also increases plant resistance to both biological and abiotic stresses. The supplement of Ca^2+^ could restrict the growth of fungal pathogen *B. cinerea* in strawberry (Langer et al., 2019). In addition, Ca^2+^ was reported to enhance resistance to *Botryosphaeria dothidea*, which is one of the most serious diseases for pear, by increasing autophagic activity and salicylic acid (SA) accumulation (Sun et al., 2020b). Transcriptome and proteome results showed that the application of Ca alleviated the temperature response of apple tree and salinity stress response of pear plants (Xu et al., 2015; Li et al., 2017).

### Molecular Pathways of Ca in Rosaceae Species

Ca^2+^ is a ubiquitous second messenger related to plant cell signaling processes and enables the development as well as biotic and abiotic stress responses in plants. The change of the intracellular free Ca^2+^ content is one of the earliest events during perceiving changes of plants in the environment. Ca^2+^ sensors calmodulin (CaM), Ca^2+^-dependent protein kinases (CDPKs), calmodulin-like proteins (CMLs), and CBLs play important roles in the process that convert Ca^2+^ signals into appropriate responses (Aldon et al., 2018). In addition to the components of the classical Ca signaling pathway, FaAnn5s and FaAnn8, plant Ca^2+^-binding proteins, were downstream of Ca signaling during strawberry fruit ripening (Chen et al., 2016). Recently, Ca^2+^/H^+^ exchangers MdCAX11 and MdCAX5, tonoplast located Ca^2+^ transport proteins, were suggested to mediate the influx of Ca from the cytosol into vacuoles, which may be related to the occurrence of BP in apple fruit, because their expression increased as the severity of BP increased (Liu et al., 2021).

A total of 4 *MdCaM* and 58 *MdCML* genes were identified in apple (*Malus* × *domestica*), and *MdCaMs*/*MdCMLs* were expressed in shoots, roots, mature leaves, flowers, and fruits (Li et al., 2019). The overexpression of *MdCML3* observably improved the salt tolerance of apple callus (Li et al., 2019). *FvCaM* and *FvCML* genes in strawberry were identified (Zhang et al., 2016). The overexpression of four *FvCaMs* and *FvCMLs* could enhance the resistance of *Nicotiana benthamiana* leaves to *Agrobacterium tumefaciens* (Zhang et al., 2016). *PbCBL1* gene, from the birch-leaf pear, is induced by gibberellic acid (GA), ABA, SA, methyl jasmonate (MeJA), and several abiotic stresses (Xu et al., 2015).

In five Rosaceae species, involving strawberry, apple, peach, pear, and plum, a total of 123 CPK genes were identified (Wei et al., 2016). Eleven out of 30 MdCPKs were regulated by pathogen infection (Kanchiswamy et al., 2013). The overexpression of *MdCIPK6L* could improve the salt tolerance in transgenic Arabidopsis and tomatoes (Wang et al., 2012). The expression of *FaCDPK* affected strawberry fruit development and ripening and also responded to salt and drought stresses, as well as ABA treatment (Crizel et al., 2020).

Transcriptome and proteome were used to determine the changes after calcium treatment during fruit development and postharvest senescence in sweet cherry (Michailidis et al., 2019, 2020). The expression of several genes involved in tricarboxylic acid (TCA) cycle, pectin degradation, as well as amino acids and sugar metabolism was affected by Ca treatment in sweet cherry (Michailidis et al., 2019). Ca specifically increased the amounts of proteins that were classified as oxidoreductases, transferases, hydrolases, lyases, and ligases during postharvest storage (Michailidis et al., 2020).

## Iron Fertilizer Improves Photosynthesis, Fruit Yield, and Quality

Iron (Fe) deficiency not only reduced biomass, chlorophyll, and photosynthesis but also increased oxidative stress response and leaf chlorosis in both peach and strawberry (Eichert et al., 2010; Gama et al., 2016; Kaya and Ashraf, 2019). However, other effects of Fe deficiency on the growth, fruit quality, and yield of different Rosaceae species were varied.

In peach, Fe deficiency led to decreased fruit production and fruit quality, because of the lower total sugar/total organic acid ratios and the less anthocyanin accumulation, and to a slight improvement of phenolic compounds and vitamin C (Alvarez-Fernández et al., 2003). It not only induced leaf chlorosis in peach but also reduced WUE, because normal stomatal functioning was disturbed in chlorotic leaves (Eichert et al., 2010).

In strawberry, deficiency of Fe resulted in a lower N assimilation, photosynthesis, and antioxidant activity, while higher amounts of bioactive compounds, including phenols and anthocyanin (Valentinuzzi et al., 2015a,b). It was shown that increased nitric oxide (NO) in the root system, root ferrous uptake activity, and indole-3-acetic acid (IAA) content in the shoot apex may enhance tolerance to Fe deficiency in *Malus xiaojinensis* and strawberry (Wu et al., 2012; Zha et al., 2014; Zhai et al., 2016; Kaya et al., 2019).

To improve the tolerance to Fe deficiency in Rosaceae plants, many measures have been taken. Studies have shown that exogenous Suc could enhance tolerance to Fe deficiency through regulating chlorophyll biosynthesis in *Malus halliana* (Guo et al., 2020). Additionally, foliar spraying with amino acid-Fe compound fertilizer remarkably increased fresh weights, Fe accumulation, total chlorophyll content, photosynthesis, and stomatal conductance of leaves in peach (*P. persica* L. Batsch; Sheng et al., 2020).

Pre-treatments with 24-epibrassinolide (EB) improved Fe deficiency tolerance *via* improving Fe^2+^ and antioxidant enzyme activities in leaves, thus causing an elevation in NR in strawberry (Kaya et al., 2020). The NO donor, sodium nitroprusside (SNP), restored Fe deficiency response in *M. xiaojinensis* (Zhai et al., 2016), and exogenously applied NO effectively reduced electrolyte leakage (EL), malondialdehyde (MDA), and H_2_O_2_ of seedlings in strawberry (*Fragaria* × *ananassa* cv. Camarosa) suffering Fe deficiency (Kaya et al., 2019).

The symptoms of Fe deficiency, such as lower chlorophyll contents in young leaves, were decreased by application of sodium hydrosulfide (NaHS), as it could improve endogenous H_2_S and antioxidant enzyme activities, as well as reduce the H_2_O_2_ and MDA generation under Fe deficiency, eventually enhancing the uptake and activation of Fe (Kaya and Ashraf, 2019).

### Molecular Pathways of Fe in Rosaceae Species

Fe is a crucial element in plants, and Fe deficiency would lead to common nutritional disorders. Fe deficiency limits the yield of fruit trees, reducing fruit quality, particularly for the pear trees growing in calcareous soil (Donnini et al., 2011). When subjected to Fe deficiency, a series of adaptive responses in plants is induced. For example, H^+^ secretion of the rhizosphere increased and led to soil acidification, promoting the conversion from Fe^3+^ to Fe^2+^, which allowed the apple tree to absorb Fe better (Zhang et al., 2020). Currently, the signal mechanism of Rosaceae responding to different Fe supplies is mainly studied in *Malus* plants.

The uptake and transport of Fe mainly depend on transporters. Fe-regulated transporter 1 (*Mx IRT1*), an Fe transporter that was highly effectively inducible in *M. xiaojinensis*, could rescue the phenotype of Arabidopsis *irt1* mutant and restore the growth defect of Fe-limit yeast mutant *DEY1453* (*fet3fet4*; Zhang et al., 2014). In genus *Malus*, a mutant allele of *IRT1* showed increased expression of *IRT1*, which allows apple to adapt to Fe deficiency (Zhang et al., 2017). In apple, 18 Fe-regulated transporter-like protein (ZIP) family genes were identified (Ma et al., 2019). MdZIP10 could not only rescue the growth of Fe^2+^ uptake defective yeast mutants but also increase the Fe content and alleviate Fe deficiency symptoms by inducing the expression of Fe uptake and transport-related genes in *MdZIP10* overexpression *A. thaliana* transgenic plants (Ma et al., 2019).

Transcription factors play important roles in Rosaceae species’ responses to Fe deficiency. In the case of Fe deficiency, *MxIRO2*, a novel basic helix-loop-helix Fe-related transcription factor, was upregulated in *M. xiaojinensis* leaves and roots (Yin et al., 2013). MdbHLH104 was identified to play a key role in toleration to Fe deficiency in both transgenic apple plants and callus by directly regulating the expression of *MdAHA8* to increase the plasma membrane (PM) H (+)-ATPase activity and Fe uptake (Zhao et al., 2016a). Additionally, MdbHLH104 was degraded by MdBT2 and MdSIZ1 (a SIZ/PIAS-type SUMO E3 ligase) dependent SUMOylation and ubiquitination through the 26S proteasome pathway in apple (*M. domestica*; Zhao et al., 2016b; Zhou et al., 2019).

Fe deficiency response transcription factor *MxFIT* was induced in roots during Fe deficiency, and the overexpression of *MxFIT* in Arabidopsis enhanced toleration to Fe deficiency, indicating that it may play roles in Fe uptake and Fe deficiency response (Yin et al., 2014). In addition, ethylene response factor MxERF4 could inhibit *MxIRT1* expression *via* binding to its promoter in *M. xiaojinensis*, which suggested that ethylene regulated the Fe deficiency response *via* MxERF4-related Fe acquisition (Liu et al., 2018). *MbERF4* and *MbERF72* were highly expressed in Fe deficiency-sensitive *Malus baccata*, whereas they were lowly expressed in Fe deficiency-tolerant *M. xiaojinensis*, and they repressed the expression of *MbHA2* directly, thus increasing the rhizosphere pH in response to Fe deficiency in *M. baccata* (Zhang et al., 2020).

With overexpressing *MdMYB58*, Fe deficiency-inducible MYB transcription factor, both apple calli and transgenic Arabidopsis could accumulate Fe under low Fe stress (Wang et al., 2018a). MdMYB58 directly repressed the expression of *MdMATE43* and *MdFRD3* in Arabidopsis, but it was competitively attenuated by MdSAT1, a member of bHLH transcription factors, through protein-protein interaction (Wang et al., 2018a). Under both deficient and normal Fe conditions, the content of Fe in transgenic Arabidopsis expressing *MxMYB1* was lower than that of wild type, that may be because of the lower expression of Fe transporter *AtIRT1* and an Fe storage protein ferritin *AtFER1* in plants (Shen et al., 2008).

Besides the transport proteins and transcription factors, MxNRAMP1 (natural resistance-associated macrophage protein), MxNas1 (nicotianamine synthase), and lncRNA MSTRG.85814.11 from *M. xiaojinensis* and *M. domestica* play important roles in Fe deficiency (Pan et al., 2015; Sun et al., 2018a, 2020a). *MxNRAMP1* overexpression could accelerate Fe absorption and accumulation and increase the resistance of plants against Fe deficiency stress (Pan et al., 2015). *MxNAS1* overexpression in transgenic tobacco cells could increase the content of active Fe and Na under Fe sufficiency, while related to the redistribution of Fe in *M. xiaojinensis* under Fe deficiency (Sun et al., 2018a). lncRNA MSTRG.85814.11 positively modulated the small auxin upregulated gene SAUR32 and increased the expression of *AHA10*, a plasma membrane proton ATPase, which activated proton extrusion involved in response to Fe deficiency (Sun et al., 2020a).

*M. halliana*, which is an apple rootstock with Fe deficiency resistance, is used to evaluate short-term molecular response under Fe deficiency treatment by RNA sequencing (RNA-Seq) analyses (Hu et al., 2018; Wang et al., 2018f). Fe deficiency induced genes involved in photosynthesis, pigment regulation, and glycolysis pathways, which suggested that Fe deficiency can also affect the synthesis and metabolism of sugar in the *M. halliana* leaves and roots (Hu et al., 2018; Wang et al., 2018f).

## Conclusion and Future Perspectives

**Figure 1:**
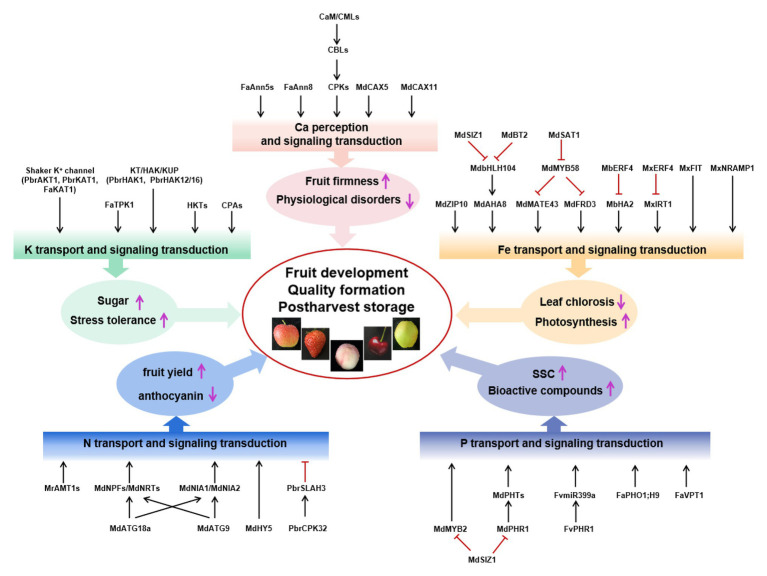
Signal components involved in the regulation of fruit yield, quality, and postharvest storage by N, P, K, Ca, and Fe. Mineral nutrients nitrogen (N; blue box), phosphorus (P; purple box), potassium (K; green box), calcium (Ca; pink box), and iron (Fe; orange box) regulate fruit development, quality formation, and postharvest storage. Black arrows represent positive interactions or regulation, whereas red bars represent repression. Ammonium transporter AMT1 in the apple rootstock *Malus robusta* Rehd, anion channel PbrSLAH3 of pear, nitrate transporter 1/peptide transporter family, NR MdNIA1/MdNIA2, and autophagy protein MdATG18a/MdATG9 from *Malus domestica* are identified in N transport or signal transduction, which mainly affect fruit yield and anthocyanin synthesis. Phosphate transporters SUMO E3 ligase MdSIZ1, MdPHR1, and MdMYB2 in apple and FaPHO1;H9, FaVPT1, FvPHR1, and FvmiR399a in strawberry are identified to change the content of soluble solid (SSC) and bioactive compounds. Shaker K^+^ channel, KT/HAK/KUP family K transporters, HKT and CPA transport proteins in five Rosaceae species, and FaKAT1 and FaTPK1 in strawberry were identified, improving sugar content and stress tolerance. MdCaM, MdCML, MdCAX5, and MdCAX11 in apple; FvCaMs, FvCMLs, FaAnn5s, and FaAnn8 in strawberry; PbCBL1 from the birch-leaf pear; and CPK family from five Rosaceae species are identified to be associated with regulating fruit firmness, keeping plants from physiological disorders, and improving sugar content and stress tolerance. Iron-regulated transporters (MxIRT1 and MdZIP), transcription factors (MxIRO2, MdbHLH104, MbERF72, MxFIT, MxERF4, MdSAT1, MdMYB58, and MxMYB1), E3 ligases (MdBT2 and MdSIZ1), and MxNRAMP1 are identified in Fe transport or signal transduction, which promoted photosynthesis and prevented leaf chlorosis.

In this review, we focused on the effect of mineral nutrition on Rosaceae species’ fruit yield, quality, and postharvest storage and, on the one hand, on the mechanisms by which Rosaceae absorbed, utilized, and transmitted nutritional signals. In conclusion, nitrogen supply mainly affects fruit yield, anthocyanin synthesis, and chlorophyll degradation in the fruit. Phosphorus (P) supplementation affects the production of SSC and secondary metabolites, such as flavonoids and ascorbic acid. Potassium (K) not only affects fruit quality but also induces both biological and abiotic stress tolerance in fruit trees. Calcium (Ca) deficiency is a major factor associated with fruit softening and BP. Iron (Fe) deficiency reduced biomass and photosynthesis, whereas it increased oxidative stress response and the activities of antioxidant enzymes. Many important transport proteins, transcription factors, and other regulators were reported and shown in Figure 1. However, there are still many significant questions to be investigated in the future.

First of all, more comprehensive understanding of the uptake and translocation system in Rosaceae species for mineral elements is required. Compared with the model plant Arabidopsis and crops, the transport and regulation system of mineral elements in Rosaceae species are far from fully understood. Although some transporters have been identified through bioinformatics, their function should be verified through more physiological and molecular experiments in the future.

Second, as the change of fertilization of some mineral nutrient elements will affect the absorption and utilization of other elements and the plants would communicate with rhizosphere microorganisms to improve the absorption and utilization of nutrients, we should pay more attention to the molecular mechanisms of the interactions between different nutrients or plants and microbes and take advantage of them.

Third, as it is difficult for the public to accept transgenic plants, especially transgenic fruits, we need to find more natural variations and refine the gene editing techniques to apply in Rosaceae, in order to improve fruit yield and quality.

## Author Contributions

YH, YS, and QB wrote the review. QB collected the references. YH designed the model and revised the review. All authors contributed to the article and approved the submitted version.

### Conflict of Interest

The authors declare that the research was conducted in the absence of any commercial or financial relationships that could be construed as a potential conflict of interest.
